# Does body mass index misclassify physically active young men

**DOI:** 10.1016/j.pmedr.2015.06.003

**Published:** 2015-06-11

**Authors:** Tyson Grier, Michelle Canham-Chervak, Marilyn Sharp, Bruce H. Jones

**Affiliations:** a Directorate of Epidemiology and Disease Surveillance, U.S. Army Public Health Command, MD, USA; b Military Performance Division, U.S. Army Research Institute of Environmental Medicine, Natick, MA, USA

**Keywords:** Body composition, Body mass index (BMI), Overweight, Military

## Abstract

**Objective:**

The purpose of this analysis was to determine the accuracy of age and gender adjusted BMI as a measure of body fat (BF) in U.S. Army Soldiers.

**Methods:**

BMI was calculated through measured height and weight (kg/m^2^) and body composition was determined by dual energy X-ray absorptiometry (DEXA). Linear regression was used to determine a BF prediction equation and examine the correlation between %BF and BMI. The sensitivity and specificity of BMI compared to %BF as measured by DEXA was calculated.

**Results:**

Soldiers (n = 110) were on average 23 years old, with a BMI of 26.4, and approximately 18% BF. The correlation between BMI and %BF (R = 0.86) was strong (p < 0.01). A sensitivity of 77% and specificity of 100% were calculated when using Army age adjusted BMI thresholds. The overall accuracy in determining if a Soldier met Army BMI standards and were within the maximum allowable BF or exceeded BMI standards and were over the maximum allowable BF was 83%.

**Conclusion:**

Using adjusted BMI thresholds in populations where physical fitness and training are requirements of the job provides better accuracy in identifying those who are overweight or obese due to high BF.

## Highlights

• The accuracy of age adjusted BMI for passing or failing body fat standards was 83%.
• The correlation between BMI and % body fat as determined by DEXA was 0.86
• An equation was derived to estimate % body fat: % body fat = 1.3974(BMI) − 19.166.
• Soldiers with a BMI of 27.5 < 30 had 10 kg more fat mass than Soldiers with a BMI < 25.
• Soldiers with a BMI of ≥ 30 had 17 kg more fat mass than Soldiers with a BMI < 25.

## Introduction

Body mass index (BMI) is uncomplicated to calculate, predictive of obesity related diseases, a simple way to monitor changes in fatness and is the recommended screening tool for overweight and obesity in large populations. The Centers for Disease Control and Prevention, the World Health Organization, and the National Heart, Lung and Blood Institute endorse or recommend BMI as a population screening tool for assessing healthy body weight (Anon., 2011a, Anon., 2013, NHLBI, 1998). As defined by the CDC and World Health Organization, a BMI of 18.5–24.9 is considered normal, 25–29.9 as overweight and 30 + is considered obese (Anon., 2011b, BMI classification, 2004).

Although recommended as a good screening tool for overweight and obesity in large populations, some investigators have suggested using caution when estimating body fat through BMI and have raised concerns about its validity (Ode et al., 2007, Wellens et al., 1996, Witt and Bush, 2005). Some of the concerns are that it cannot differentiate between lean body mass and fat mass, and the distribution of fat over the body is not captured. The BMI classification system (Anon., 2011b) is based on observational and epidemiological studies relating BMI to the risk of morbidity and mortality (Hamm et al., 1989, Gordon and Doyle, 1988, Hubert et al., 1983, Rabkin et al., 1977, Lipton et al., 1993, Colditz et al., 1990). However it has been stated that the cut points obtained from these general population studies may not be specific to subgroups of athletes and young non-athletes (Ode et al., 2007).

As rates of obesity rise, there is growing interest in the measurement of body composition, especially the assessment of fat mass. Screening for body composition is of high interest because of the potential health consequences associated with excessive body fat such as diabetes, hypertension and cardiovascular disease (Hubert et al., 1983, Colditz et al., 1990, Black et al., 1983). Others such as the military and emergency service personnel may also have an interest in body composition due to its relation with physical performance. In the United States adult population, obesity as determined by BMI has more than tripled from 11% in 1960 to 36% in 2010. Those classified as overweight have remained fairly constant over the last 50 years at approximately 33% (Anon., 2012b). However, this distribution is not reflective of the U.S. Army population. In the U.S. Army, as of 2011, 52% of service members were considered overweight, whereas only 16% were considered obese (DOD, 2011).

There are multiple techniques or methods used to assess body fat. Multicomponent models which distinguish between fat and fat free masses are considered as referent or criterion methods against which other methods are evaluated (Wells & Fewtrell, 2006). Some examples of referent methods include underwater densitometry and dual energy X-ray absorptiometry (DEXA). However, the use of these techniques can be time consuming, expensive and not practical when evaluating large populations. A more practical approach to measuring body fat in large populations would be prediction methods, where body fat is estimated from specific or population based equations. Some examples of prediction methods include skinfold measurements, bioelectrical impedance and anthropometric methods such as BMI. The validity of BMI has been compared to these and other body fat measurement techniques (Ode et al., 2007, Wellens et al., 1996, Witt and Bush, 2005, Garrow and Webster, 1985). The correlations between percent body fat as measured by referent or prediction methods and BMI have ranged from 0.62 to 0.96 (Ode et al., 2007, Wellens et al., 1996, Garrow and Webster, 1985, Gupta and Satwanti, 2014, Hakkinen et al., 2010, Gallagher et al., 2000, Deurenberg et al., 1991).

The Army height–weight tables are age and gender adjusted and based on BMI used as an estimator of body fat. Some concerns of service members are that the methods used to estimate body composition not only identify over-fat Soldiers but also Soldiers of more muscular builds as being overweight. The purpose of this analysis was to assess the accuracy of age and gender adjusted BMI as a measure of body fat in a sample of U.S. Army infantry Soldiers.

## Methods

This investigation is a secondary analysis of previously collected data by the U.S Army Research Institute of Environmental Medicine (USARIEM) and the U.S. Army Public Health Command (USAPHC). The study was approved by the USARIEM Human Use Review Committee. There were 110 male U.S. Army Soldiers from an infantry battalion within the United States who volunteered to participate and signed a volunteer agreement after being briefing about the study. Human subjects participated in these studies after giving their free and informed voluntary consent. Investigators adhered to the Department of Defense Instruction 3216.02 and the title 32 Code of Federal Regulations part 219 on the use of volunteers in research.

All measurements were taken 1–2 months before the battalion was scheduled for deployment. Demographic data such as age, race, education level, and military occupational specialty were obtained through rosters and the Defense Medical Surveillance System.

Height and weight were measured, respectively, using stadiometers (Portable Height Rod, Seca Scales, Hamburg, Germany) and digital scales, (Seca Alpha Model 770, Seca Scales) with the Soldiers in physical training shirts, shorts and socks. From the measured height and weight, BMI was calculated as weight in kilograms (kg) divided by height in meters (m) squared (kg/m^2^). When examining BMI by average lean body mass, fat mass and % body fat, those in the overweight BMI category (BMI 25–29.9) were split into a low overweight group (25–27.49) and a high overweight group (27.5–29.99) (BMI classification, 2004) The WHO states that a cut point of 27.5 can be added as a point for public health action (BMI classification, 2004). Total lean body mass (LBM) and fat mass (FM) differences were calculated using the normal or healthy weight group (BMI < 25) as the reference. Body composition was measured by a Hologic model QDR 4500 W Dual-Energy X-ray Absorptiometry (DEXA) densitometer (Hologic Inc., Bedford, MA). Hologic software algorithms provided estimates of percent body fat, absolute body fat, total fat-free mass, bone mineral content, and bone mineral density. Soldiers were positioned supine on the DEXA table with their arms at their sides and their feet strapped down. The accuracy of DEXA has been reported to be within 1–3% of other multicomponent models (Lohman et al., 2000).

The Statistical Package for the Social Sciences (SPSS®), Version 19.0, was used for statistical analysis. Descriptive statistics (frequencies, distributions, means, standard deviations (SD)) were calculated for personal characteristics and body composition. The sensitivity and specificity of BMI were calculated in comparison to % body fat (BF) as determined by DEXA. This analysis used the following cut-points taken from Army Regulation (AR) 600-9 (The Army Body Composition Program Publication), which specifies allowable body fat percentages derived from BMI thresholds by age group: < 21 years/BMI 25.9/BF 20%; 21–27 years/BMI 26.5/BF 22%; 28–39 years/BMI 27.2/BF 24%; and > 40 years/BMI 27.5/BF 26%. The analysis also examined specific BMI cut points of ≤ 25 and the optimal BMI cut point of ≤ 28.3 as calculated by Youden's index. Youden's index which is often used in conjunction with the Receiver Operating Characteristic (ROC) analysis maximizes sensitivity and specificity to determine an optimal cut-point (Ruopp et al., 2008, Schisterman et al., 2005). Linear regression was used to determine a body fat prediction equation and examine the correlation between % body fat and BMI. A power estimation was performed on the 110 subjects using an alpha of .025 and a correlation coefficient of .80.

## Results

The average age of the Soldiers in the battalion was 23 ± 4.5 years with an age range of 18 to 43 years. A majority of the Soldiers were Caucasian, high school graduates, and had a military occupational specialty (MOS) of infantryman. The descriptive statistics are displayed in Table 1.

Physical characteristics are displayed in Table 2. Soldiers had a mean BMI of 26.4, percent body fat of 17.5%, and 63 kg of lean body weight.

**Fig. 1:**
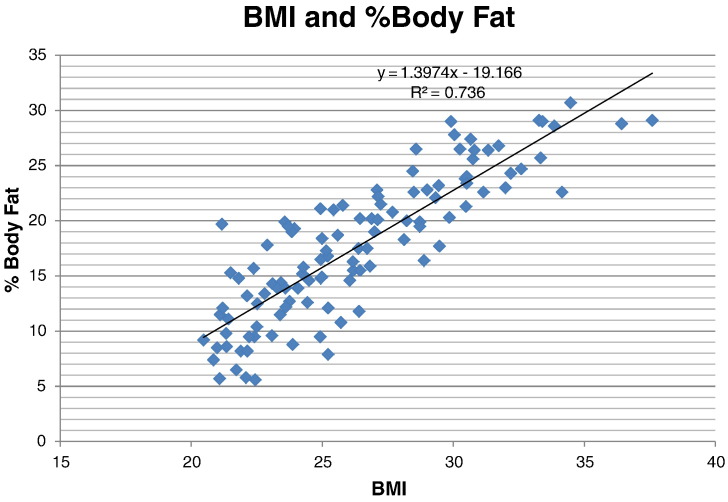
Correlation between BMI and % body fat as measured by DEXA (r = 0.86).

Table 3 displays BMI, lean body mass, fat mass and the average percent body fat associated with the corresponding BMI. In the low overweight group, Soldiers on average had 5.5 kg more LBM and 5 kg more FM than Soldiers in the normal group, representing a percent difference of 9% and 55% respectively. In the high overweight group, Soldiers on average had 7.5 kg more LBM and 10 kg more FM than Soldiers in the normal group, representing a percent difference of 13% and 110% respectively. In the obese group, Soldiers on average had 12.1 kg more LBM and 17 kg more FM than Soldiers in the normal group, representing a percent difference of 21% and 187%, respectively. DEXA data indicated that percent body fat increased linearly (p < 0.01) and was almost double for the obese group, when compared to the normal group (Table 3 and Fig. 1). Overall FM is more than double in the high overweight and obese group compared to the normal group while LBM is only 13% and 21% higher among high overweight and obese Soldiers, respectively.

The correlation between BMI and % body fat as determined by DEXA was 0.86 (p < 0.01) (Fig. 1) and the following equation was derived to estimate percent body fat: % body fat = 1.3974(BMI) − 19.166. The power to obtain a correlation coefficient with 110 Soldiers of .80 was 100%.

The accuracy of this equation is ± 3.3% when compared to the body fat measurements determined by DEXA. Using this prediction equation for Soldiers in the current study estimates average body fat at 17.7%.

Table 4 displays sensitivity, specificity and correlations between BMI and % body fat. A sensitivity and specificity of 77% and 100% were calculated when using established BMI thresholds as determined by age group. The sensitivity and specificity were also calculated for BMI cutoff points of 25 and the optimal cutoff point of 28.3 as determined by Youden's index. (Table 4.)

Table 5 displays the number of Soldiers meeting or exceeding the age adjusted BMI Standards and if they passed or failed their maximum allowable body fat as determined by AR 600-9. The overall accuracy in determining if a Soldier met the BMI standards for their age group and were within their maximum allowable body fat or exceeded their BMI standards and were over their maximum allowable body fat was 83%. The 19 Soldiers who exceeded BMI thresholds, yet passed body fat standards were on average within 2.7% of the maximum allowable body fat.

## Discussion

The purpose of this study was to assess if BMI is a valid measure of body fat in U.S. Army Soldiers. The correlation between BMI and body fat as measured by DEXA was strong (Taylor, 1990) (0.86) and accounted for 74% of the variability. It was shown that Soldiers in the overweight and obese categories had additional lean body mass. However, this additional lean body mass was also accompanied by a disproportionate % increase in additional body fat for the overweight and obese groups. When looking at the overall accuracy of BMI as a prediction tool in estimating body fat, it accurately identified 83% of the Soldiers as within or over the maximum allowable Army age adjusted body fat standards. The prediction equation which is specific to infantrymen (% body fat = 1.3974(BMI) − 19.166) also demonstrated good accuracy by predicting body fat within ± 3.3% of the DEXA body fat measurements.

There was a strong correlation shown between BMI and % body fat as measured by DEXA (r = 0.86). Other studies have indicated moderate to very strong correlations between BMI and % body fat (0.65–0.95) as measured by referent and predictive methods (Ode et al., 2007, Wellens et al., 1996, Witt and Bush, 2005, Garrow and Webster, 1985, Gupta and Satwanti, 2014, Hakkinen et al., 2010, Gallagher et al., 2000, Deurenberg et al., 1991). Similar to the current study, Gallagher et al. performed a study to develop prediction equations derived from DEXA and other variables such as age and gender to predict % body fat using BMI. Gallagher's prediction equation, that combined Caucasian and African American data to predict % body fat, had a correlation of 0.86, the same as the current study (Gallagher et al., 2000). Using Gallagher's equation and the current study's BMI data, average % body fat was estimated to be 19.7%. Using the current study's prediction equation and BMI data, average body fat was estimated at 17.7%. Therefore, Gallagher's equation slightly overestimated body fat for the Soldiers by approximately 2%. However, this would be expected since Gallagher's equation is based on the general population (1626 healthy adults with BMI's ≤ 35) and not military personnel. It would seem that even though both correlations were strong, prediction equations are more accurate for the population in which they were derived. Other studies that have indicated strong correlations between BMI and % body fat have suggested using caution when interpreting BMI and fatness in athletes and military personnel (Ode et al., 2007, Wellens et al., 1996, Witt and Bush, 2005, Prentice and Jebb, 2001). They generally state that BMI overestimates body fat in specific groups such as military personnel, athletes, male firefighters, and other specific groups requiring or having additional muscle mass. It has been stated that these more muscular individuals are just seen as ‘noise’ within a linear regression performed on the general population (Prentice & Jebb, 2001). The current study suggests otherwise, showing that overweight and obese Soldiers display a disproportionate increase in fat compared to lean mass.

When examining the sensitivity and specificity of the Army age adjusted BMI thresholds (25.9–27.5) and the CDC overweight and obese categories (BMI ≥ 25), the sensitivity was 17% higher for the Army standards (77% vs. 60%, respectively), while the specificity was the same (100%). However, sensitivity and specificity were maximized when using the BMI threshold cut point of 28.3 as determined by Youden's index (Table 4). The Army currently uses a maximum BMI threshold of 27.5 for men over 40 years old based on chronic health disease risk and a corresponding waist circumference of 38.5 in. (Friedl, 2012). In a study of college athletes and non-athletes from a university, BMI was also assessed as a predictor of body fat. Body fat was determined by densitometry via the Bod Pod. In this study specificity was highly improved when using optimal cut-points for BMI of 27.9 (men) and 27.7 (women) for college athletes and 26.5 (men) and 24.0 (women) for college non-athletes. The author concluded that BMI should be used with caution when classifying college athletes and non-athletes as over fat and that the results support a need for a different classification system of overweight in these populations (Ode et al., 2007). In another study investigating obesity, they also found that adjusted BMI cut off points, had higher sensitivity and specificity than when using published recommendations (Anon., 2011b, Wellens et al., 1996).

One of the biggest concerns of using BMI to estimate body fat is that it will unfairly classify Soldiers with greater lean body mass as being overweight or obese. In this investigation, overweight and obese Soldiers had an additional 5–12 kg of muscle mass. However, this additional lean body mass was also accompanied by 5–17 kg of fat mass. There was approximately 1.3–1.4 kg of additional fat gained for every 1 kg of lean body mass in the high overweight and obese groups. In the overweight and obese groups, overweight and obese Soldiers appear to have more lean body mass, but also have a disproportionally greater percent of body fat compared to Soldiers in the normal BMI category.

When comparing the BMI's of the Soldiers in the current evaluation to the overall Army population, there were more Soldiers in the healthy weight group (45% vs. 28%), less Soldiers who were overweight (35% vs. 54%) and more Soldiers who were considered obese (21% vs. 17%) (DOD, 2011). However, the distribution of lean body mass and fat mass in an average Army infantryman is not known. Nonetheless, other studies have examined body composition as measured by DEXA in different populations (Gallagher et al., 2000, Michell et al., 2014). In an investigation of Italian Navy seal recruits, recruits were about 13% body fat with an average of 69 kg of lean body mass and 9 kg of fat mass, as measured by DEXA. Their average BMI was 25.4. In the current study the group with a BMI ≥ 30 had on average 70 kg of lean body mass, similar to the Italian Navy seal recruits. Although the Italian Navy seal recruits had about half the body fat (13% vs. 25%) (Malavolti et al., 2008). It appears that overweight and obese Infantrymen in the current study have higher BMI's due to more body fat as opposed to lean body mass.

Eighty three percent of the Soldiers were correctly identified using the age adjusted BMI thresholds as meeting or exceeding the Army body fat standards. The 17% of Soldiers identified as exceeding BMI thresholds, but passing their body fat standards were on average within 2.7% of the maximum allowable body fat. Therefore these Soldiers were close to meeting or exceeding the maximum allowable % body fat for their age. This cannot be attributed to just high levels of lean body mass. In a study of Belgian male military candidates (n = 448) they investigated the validity of BMI as a measure of body fat through bioelectrical impedance. They also showed that 83% of the candidates were identified correctly as either normal weight (BMI < 25, % body fat < 21%) or overweight (BMI ≥ 25, % body fat ≥ 21%) (Mullie et al., 2008). In studies investigating the validity of BMI and body fat in firefighters (who also have physically demanding occupations), 60–79% of the firefighters were identified correctly (Jitnarin et al., 2012, Ode et al., 2014). The results from these studies are similar to the findings from the current study. The literature and the current study indicate that BMI is a reasonable screening tool for detecting overweight and obese individuals. It should be kept in mind that for those Soldiers who are identified as overweight or obese by age and gender adjusted BMIs that there is an additional screening step for validation prior to any corrective action being taken.

## Conclusion

In general BMI is a valid and easy to use screening tool in identifying those who are overweight or obese. In specific populations where physical fitness and training are requirements of the job, then adjusting BMI cut points to optimal levels provides better accuracy in identifying those who are overweight or obese due to high body fat. It would seem that the U.S. Army age adjusted BMI threshold cut points are effective in estimating body fat and determining those who are overweight. Data from the current study suggests that the majority of overweight and obese Soldiers were classified correctly. Although, there will always be a small percentage of Soldiers who are lean and carry additional muscle mass exceeding their BMI thresholds, it should also be kept in mind that BMI is only the first step in the Army's process to identify Soldiers who are overweight or obese.

Citations of commercial organizations and trade names in this report do not constitute an official Department of the Army endorsement or approval of the products or services of these organizations.

The views expressed in this paper are those of the author(s) and do not necessarily reflect the official policy of the Department of Defense, Department of the Army, U.S. Army Medical Department or the U.S.

## Conflict of interest statement

The authors declare that there are no conflicts of interest.

## Figures and Tables

**Table 1:** Demographic data for the study sample.

Variable	Category of variable	n (%)
Gender	Men	110 (100%)
Age	< 21 years	34 (31%)
21–24 years	52 (47%)
25 + years	24 (22%)
Race	Caucasian	81 (74%)
Asian	3 (3%)
Black	9 (8%)
Hispanic	10 (9%)
Other	7 (6%)
Education level	GED	14 (13%)
High school	66 (60%)
Some college	25 (23%)
College grad or higher	3 (3%)
Graduate degree	2 (2%)
MOS	11A (infantry officer)	1 (1%)
11B (infantrymen)	88 (80%)
11C (indirect fire infantrymen)	9 (8%)
13F (fire support specialist)	1 (1%)
25U (signal support systems specialist)	3 (3%)
56M (chaplain assistant)	1 (1%)
91W (health care specialist)	7 (6%)

**Table 2:** Physical characteristics and physical training for men in the infantry battalion.

Variable	Level of variable	Men	Mean ± SD	Range (min–max)
n (%)
Height	< 173 cm	28 (26%)	177.5 ± 6.9	160–201
173–177 cm	28 (26%)
178–181 cm	26 (24%)
> 181 cm	28 (26%)
Weight	< 71 kg	27 (25%)	83.3 ± 14.7	56–130
71–82 kg	31 (28%)
83–91 kg	25 (23%)
> 91 kg	27 (25%)
BMI	< 25	49 (45%)	26.4 ± 3.9	20.5–32.0
25–29.9	38 (35%)
≥ 30	23 (21%)
% body fat	< 13%	29 (26%)	17.5 ± 6.3	5.6–29.1
13–16%	23 (21%)
17–21%	27 (25%)
22%+	31 (28%)
Lean body mass	40–57 kg	28 (26%)	62.8 ± 7.3	40.7–78.8
58–62 kg	24 (22%)
63–68 kg	33 (30%)
69–79 kg	25 (23%)

**Table 3:** BMI by average lean body mass, fat mass and % body fat.

BMI	n	Lean body mass (LBM) (kg)	% Difference calculated from LBM compared to a BMI of < 25	Fat mass (FM) (kg)	% Difference calculated from FM compared to a BMI of < 25	% Body fat (DEXA)
< 25 (Normal)	49	58.1 ± 6.2 kg	—	9.1 ± 3.3 kg	—	12.7 ± 4.1%
25 < 27.5 (Low overweight)	23	63.6 ± 4.7 kg	+ 5.5 kg (9%)	14.1 ± 3.7 kg	+ 5 kg (55%)	17.2 ± 3.9%
27.5 < 30 (High overweight)	15	65.6 ± 4.5 kg	+ 7.5 kg (13%)	19.1 ± 3.0 kg	+ 10 kg (110%)	21.6 ± 3.4%
≥ 30 (Obese)	23	70.2 ± 5.6 kg	+ 12.1 kg (21%)	26.1 ± 3.9 kg	+ 17 kg (187%)	25.2 ± 3.5%

**Table 4:** Sensitivity, specificity and correlation of BMI with % body fat and results of other investigations.

n	BMI compared to % body fat as determined by:	BMI reference point (kg/m^2^)	Sensitivity	Specificity	Correlation with % body fat
Men n = 110	Dual-energy X-ray absorption	25.9–27.5	77%	100%	0.86
Men n = 110	25 (use 25 for all age groups)	60%	100%
Men n = 110	28.3 (use 28.3 for all age groups)	90%	96%

^a^ The Army Regulation AR 600-9 uses tabled values rounded from BMI thresholds. Thresholds of 25–27.5 are permitted (DoDI 1308.3, DoD Physical Fitness and Body Fat Program Procedures, November 5, 2002). The values below are for men:

< 21 years/BMI 25.9/BF 20%.

21–27 years/BMI 26.5/BF 22%.

28–39 years/BMI 27.2/BF 24%.

> 40 years/BMI 27.5/BF 26%.

**Table 5:** Soldiers meeting or exceeding age adjusted BMI standards and body fat as determined by AR 600-9.

Age group and BMI threshold and maximum allowable body fat (AR 600-9)	n	True positive	True negative	False negative	True positive + true negative/n
Under BMI and passed body fat	Over BMI and failed body fat	Over BMI and passed body fat	Using BMI only, % soldiers correctly identified as meeting or exceeding army body fat standards
< 21 years (25.9 kg/m^2^) (20% BF)	34	23	5	6	82%
21–27 years (26.5 kg/m^2^) (22% BF)	63	35	19	9	86%
28–39 years (27.2 kg/m^2^) (24% BF)	11	4	4	3	73%
> 40 years (27.5 kg/m^2^) (26% BF)	2	1	0	1	50%
Total	110	63	28	19	83%
